# Clinical Study on Middle Ear Diseases in Children with Orofacial Clefts

**DOI:** 10.7759/cureus.2175

**Published:** 2018-02-08

**Authors:** Raja Kalaiarasi, Kamran A Syed, Chellappa Vijayakumar, Ajoy M Varghese, Mary John

**Affiliations:** 1 Otorhinolaryngology, Sri Lakshmi Narayana Institute of Medical Science, Puducherry, India; 2 Otorhinolaryngology, Christian Medical College Hospital, Vellore, India; 3 Surgery, Jawaharlal Institute of Postgraduate Medical Education and Research (JIPMER), Puducherry, India.

**Keywords:** cholesteatoma, otitis media with effusion, middle ear effusion, pure-tone audiometry, tympanometry

## Abstract

Introduction

Orofacial clefts are associated with middle ear diseases, but the magnitude of this problem is not generally well appreciated. The aim of this study was to describe the middle ear findings and audiological profile in children with orofacial clefts.

Materials and methods

Children with orofacial clefts attending plastic surgery and otorhinolaryngology departments of a tertiary hospital over one-year duration were included in this study. Ninety-six children with orofacial clefts were identified. They were categorized age-wise as zero to five years, more than five years to 10 years, and more than 10 years to 15 years. They underwent a detailed ear, nose, and throat examination followed by audiological tests, including brainstem evoked response audiogram (BERA), pure tone audiogram (PTA), and tympanometry.

Results

Among 96 children with orofacial clefts, only 24 children (25%) had symptomatic ear problems, whereas on ear examination, 56 children (58.3%) had abnormal ear findings. Middle ear effusion (MEE) was the most common ear condition, and it was seen in 94 ears (48.9%). Cholesteatoma was noted in six children (3.1%). Out of 73 children in the zero to five age group, 58 children (79.5%) did not have any history of ear problem but 55.5% (81 ears) had features of MEE such as a dull tympanic membrane (TM). In the age group of more than five years to 10 years, only four children (28.5%) were symptomatic. Five children (55.5%) out of nine in the age group of more than 10 years had a symptomatic ear problem of which four children (44.4%) had chronic otitis media squamosal disease. The earlier age groups showed a trend of ear disease being asymptomatic compared to older children. Normal hearing was present in only 40 children (41.7%) and various degrees of hearing loss were seen in 56 children (58.3%). The mean air conduction threshold in the age group zero to five years, more than five years to 10 years, and more than 10 years were 33±8.3 decibels (dB), 25±6.2 dB, and 31.5±14 dB, respectively. Out of 144 ears (72 children) in the asymptomatic group, 67 ears (46.5%) had normal hearing. Seventy-seven ears (53.5%) had some degree of hearing loss. Mean air conduction hearing loss in the asymptomatic group was 29.8±7.3 dB.

Conclusion

This study highlights the higher incidence of middle ear diseases compared to the presenting symptoms in children with orofacial clefts. This stresses on the need for a detailed otological evaluation to identify any middle ear pathology so that timely intervention can be taken.

## Introduction

The orofacial cleft is the most common birth anomaly affecting approximately one in 700 births [1]. In this condition, due to incomplete embryological closure, difficulties arise with feeding, speech, language, dentition, and hearing. Middle ear diseases are known to be associated with orofacial clefts, but the prevalence and effect of this problem are usually underestimated. A lot of attention has been paid to repair the facial deformity in these children. Unfortunately, when the lip and palatal deformities are investigated and managed, hearing loss, which occurs as a complication of the associated middle ear disease, sometimes gets ignored. The literature reports a higher incidence of middle ear pathologies and hearing loss in children with orofacial clefts [2-3]. The present study was conducted to describe the middle ear diseases in children with orofacial clefts and to assess their audiological status.

## Materials and methods

This study included all children with orofacial clefts presenting to the plastic surgery and otorhinolaryngology outpatient department (OPD) for a one-year duration at a tertiary teaching center. The participants were recruited in the study after obtaining written consent from their parents or guardians. The children were grouped into three categories according to age: zero to five years, more than five years to 10 years, and more than 10 years to 15 years. All the subjects underwent a detailed ear, nose, and throat examination, including an examination of the ear under the microscope. Hearing was assessed using audiological tests that included pure tone audiogram (PTA) or brainstem evoked response audiogram (BERA), and tympanometry with acoustic reflex. Older children underwent PTA using the Grason Stadler audiometer (Grason-Stadler Inc., MN, US) at frequencies 500, 1000, and 2000 Hz. For children five years or younger and uncooperative children, a hearing assessment was done by BERA using the Intelligent Hearing Screening machine (Intelligent Hearing Systems, FL, US). In BERA, a click stimulus from 60 to 90 dB above the hearing threshold in the frequency 2000 Hz to 4000 Hz and tone bursts were used. The stimulus rate used was 30.1 clicks/sec. Tympanometry was done in all children to assess the middle ear using the Grason Stadler 61 tympanometer with a probe frequency of 678 Hz. The degree of hearing loss was categorized using the American Speech Language Hearing Association (ASHA) classification [4]. The Nagpur classification system was used for describing orofacial clefts in children [5]. Group I is cleft of lip, group I (A) is cleft of lip with alveolus, group II is cleft of palate alone, group II (S) is submucous cleft of palate, and group III is cleft of lip and palate. The side of cleft was mentioned as (R) for right, (L) for left, and (B/L) for bilateral. Sade's classification was used to describe pars tensa retractions of the tympanic membrane (TM). Grade I is mild retraction, grade II is retraction onto incudostapedial joint, grade III is retraction onto promontory, and grade IV is adhesion of pars tensa to the medial wall of the middle ear.

The sample size was calculated using Master software version 2.0 (CMC, Vellore, India). Statistical inputs were taken from the previous study by SY Khan et al. Keeping the expected proportion as 81.8% and absolute precision as eight, the sample size was calculated as 90 [2]. To account for an expected 10% loss to follow-up, the sample size was inflated to 96.

## Results

Age and gender distribution

**Figure 1:**
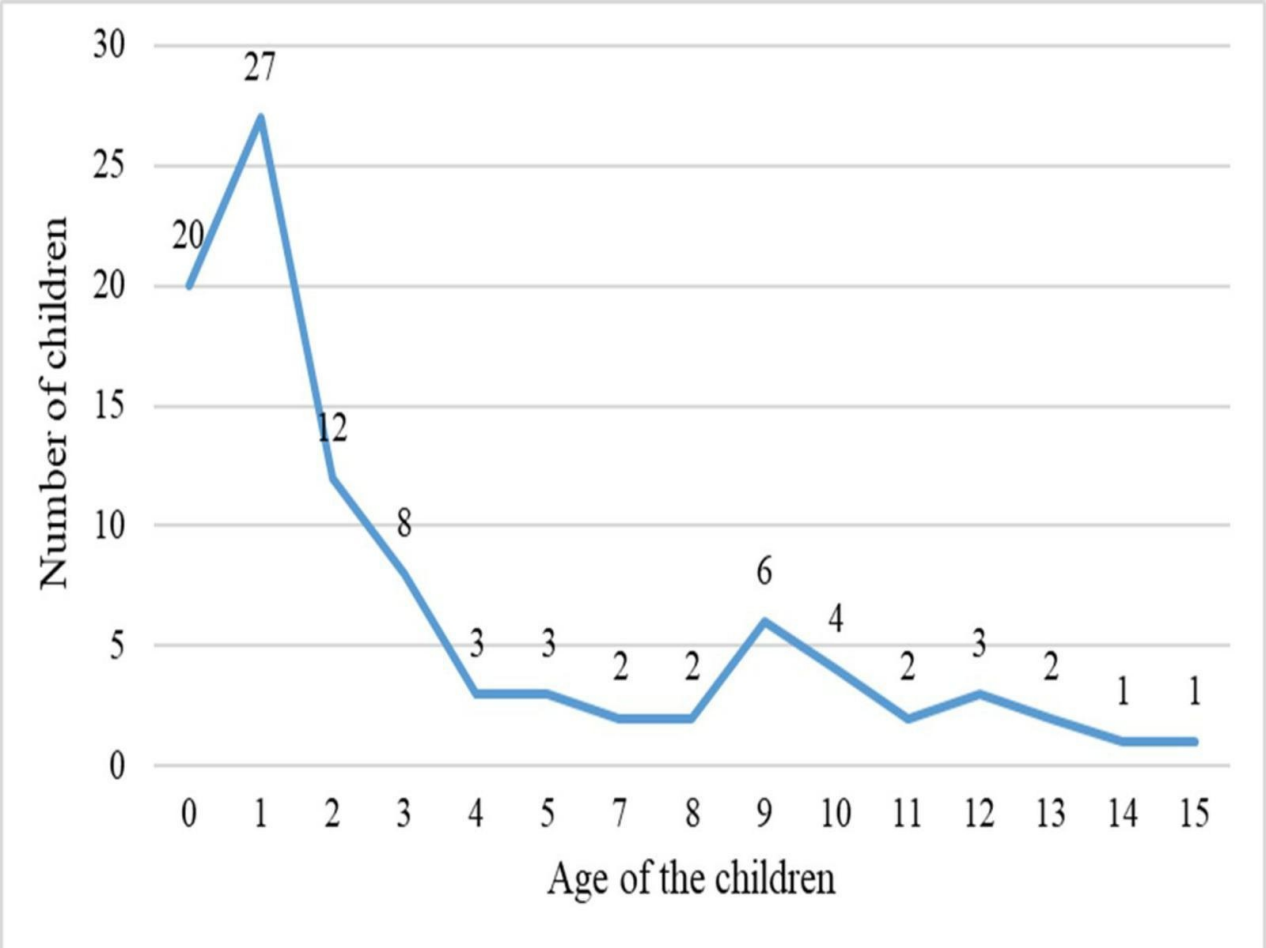
Distribution of age in children with orofacial clefts

A total of 96 children with orofacial clefts were studied. The mean age of the study population was 3.8 years. The youngest child's age in the study was four months and the oldest child was 15 years (Figure 1).

The subjects were predominantly female, with a ratio of 1.5:1. The maximum number of children was found in the age group zero to one year.

Ear symptoms and findings in the study population

Out of 96 children with the orofacial cleft, only 24 children (25%) had symptomatic ear problems (Table 1).

**Table 1:** Distribution of ear symptoms in the study population

S.No.	Ear symptoms	No. of children with orofacial clefts (%)
1.	No ear complaints	72 (75%)
2.	Hard of hearing	13 (13.5%)
3.	Ear discharge	6 (6.2%)
4.	Recurrent ear pain	5 (5.2%)

The most common ear finding noted was a dull TM that was seen in 94 ears (48.95%). Normal ear findings were seen in 98 ears (51%). Out of 73 children in the zero to five age group, 58 (79.5%) children did not have any history of ear problem but 55.5% (81 ears) had features of Middle Ear Effusion (MEE) like a dull TM. In the age group more than five years to 10 years, only four (28.5%) children were symptomatic. Five (55.5%) children out of nine in the age group more than 10 years had symptomatic ear problem of which four (44.4%) children had chronic otitis media squamosal disease. Age wise-ear symptoms and findings were described (Table 2).

**Table 2:** Ear symptoms and signs in three study groups of children TM - Tympanic membrane TS - Tympanosclerosis yrs - years

No.	Study groups [No. (%)]	Symptoms [No. (%)]	Findings [No. (%)]
1.	0-5 yrs [73 (76%)]	Hard of hearing [11 (15%)]	Dull TM [81 (55.5%)]
		Pain [3 (4.1%)]	
		Discharge [1 (1.4%)]	Normal TM [65 (44.5%)]
		Asymptomatic [58 (79.5%)]	
2.	> 5 to 10 yrs [14 (14.6%)]	Discharge [3 (21.4%)]	Dull TM [9 (32.14%)]
			Grade IV TM [3 (10.7%)]
		Hard of hearing [1 (7.14%)]	Grade IV TM [1 (3.6%)]
		Asymptomatic [10 (71.3%)]	Congested TM [1 (3.6%)]
			Normal TM [14 (50%)]
3.	> 10 to 15 yrs [9 (9.4%)]	Pain [2 (22.2%)]	Dull TM [4 (22.2%)]
		Hard of hearing [1 (11.1%)]	Grade IV TM [3 (16.7%)]
		Discharge [2 (22.2%)]	Grade II TM [1 (5.5%)]
		Asymptomatic [4 (44.4%)]	TS [2 (11.1%)]
			Normal TM [8 (44.4%)]

The most common orofacial cleft seen in this study group was the cleft palate alone (group II cleft) in 41 (42.7%) children followed by cleft of lip and palate (group III cleft) seen in 31 (32.3%) children (Table 3).

**Table 3:** Ear findings in different type of orofacial clefts TM- Tympanic membrane TS- Tympanosclerosis

No.	Type of Clefting [No. (%)]	Symptoms [No. (%)]	Findings [No. (%)]
1.	Group I [8 (8.3%)]	Asymptomatic [8 (100%)]	Normal TM [16 (100%)]
2.	Group I (A) [8 (8.3%)]	Asymptomatic [8 (100%)]	Normal TM [16 (100%)]
3.	Group II [41 (42.7%)]	Pain [10 (24.4%)]	Dull TM [48 (58.9%)]
		Hard of hearing [4 (9.8%)]	Grade IV TM [3 (3.6%)]
		Discharge [4 (9.8%)]	Grade II TM [1 (1.2%)]
		Asymptomatic [23 (56%)]	TS [2 (2.4%)]
			Normal TM [28 (33.7%)]
4	Group II (S) [8 (8.3%)]	Asymptomatic [8 (100%)]	Dull TM [6 (37.5%)]
			Normal TM [10 (62.5%)]
5	Group III [31 (32.3%)]	Pain [1 (3.2%)]	Dull TM [40 (64.5%)]
		Hard of hearing [3 (9.87%)]	Grade IV TM [3 (4.8%)]
		Discharge [2 (6.4%)]	Congested TM [1 (1.6%)]
		Asymptomatic [25 (80.6%)]	Grade II TM [1 (1.2%)]
			Grade IV TM [1 (1.2%)]
			Normal TM [17 (27.4%)]

Audiogram findings

BERA was done on 68 patients (136 ears), majority of the patients had mild hearing loss, which was seen in 39 ears (28.7%). Normal hearing was seen in 54 ears (39.7%) followed by minimal hearing loss in 20 ears (14.7%). PTA was done in 28 children (56 ears). Majority of the children (26 ears, 46.4%) had normal hearing followed by minimal hearing loss in 12 ears (21.4%). The audiological status of the study population was described (Table 4).

**Table 4:** Brainstem evoked response audiogram and pure tone audiogram findings in the study population BERA- Brainstem Evoked Response Audiogram PTA- Pure Tone Audiogram

S.No.	Degree of hearing loss	BERA [No. (%)]	PTA [No. (%)]
1.	Normal hearing	54 (39.7%)	26 (46.4%)
2.	Minimal hearing loss	20 (14.7%)	12 (21.4%)
3.	Mild hearing loss	38 (27.9%)	8 (12.5%)
4.	Moderate hearing loss	15 (11%)	6 (10.7%)
5.	Moderately severe hearing loss	-	-
6.	Severe hearing loss	3 (2.2%)	-
7.	Profound hearing loss	6 (4.4%)	4 (7.14%)

The mean air conduction threshold in the age group zero to five years, more than five years to 10, and more than 10 years were 33±8.3 decibels (dB), 25±6.2 dB and 31.5±14 dB respectively.

Tympanogram findings

Tympanogram was done in 90 children (180 ears), type A curve was seen in 81 ears (45%) followed by type B curve in 65 ears (36.1%). Tympanometry could not be done in six children as proper seal could not be achieved (Table 5).

**Table 5:** Tympanogram findings in the study population

S.No.	Types of tympanogram curves	No. of ears (%)
1.	A	81 (33.9%)
2.	As	27 (16%)
3.	Ad	2 (1.2%)
4.	B	65 (38.6%)
5.	C	5 (3%)

Acoustic reflex was tested in 90 (180 ears) children and reflex was present in 74 ears (41.1%) and absent in 106 ears (58.9%).

Audiogram and tympanogram findings in the asymptomatic children with orofacial clefts

**Figure 2:**
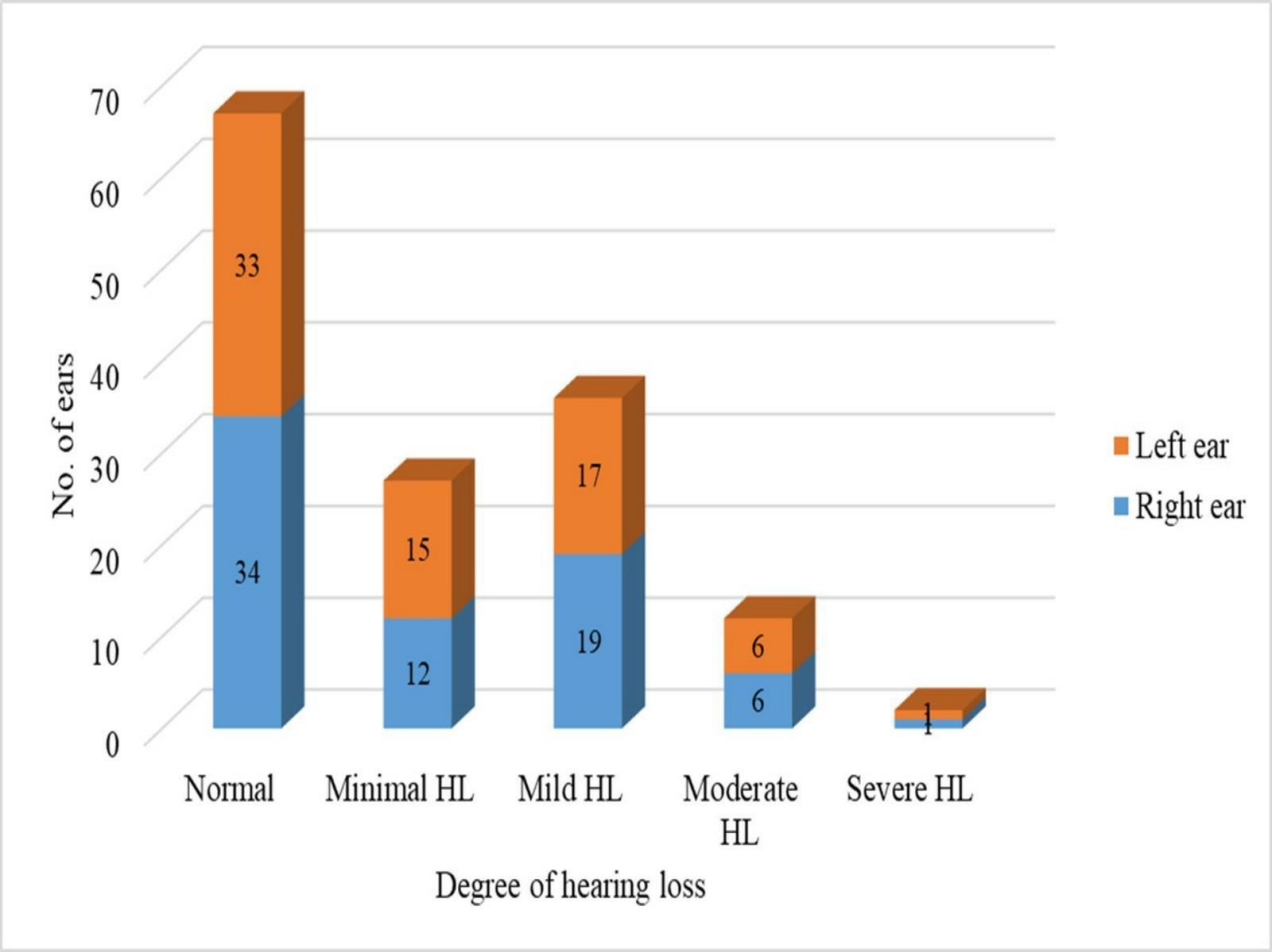
Audiogram findings in the asymptomatic group showing the degree of hearing loss (HL)

Out of 144 ears (72 children) in the asymptomatic group, 67 ears (46.5%) had normal hearing. A total of 77 ears (53.5%) had some degree of hearing loss. The mean air conduction hearing loss in the asymptomatic group was 29.8±7.3 dB. Audiogram findings of children with orofacial clefts without any history of ear symptoms were shown (Figure 2).

**Figure 3:**
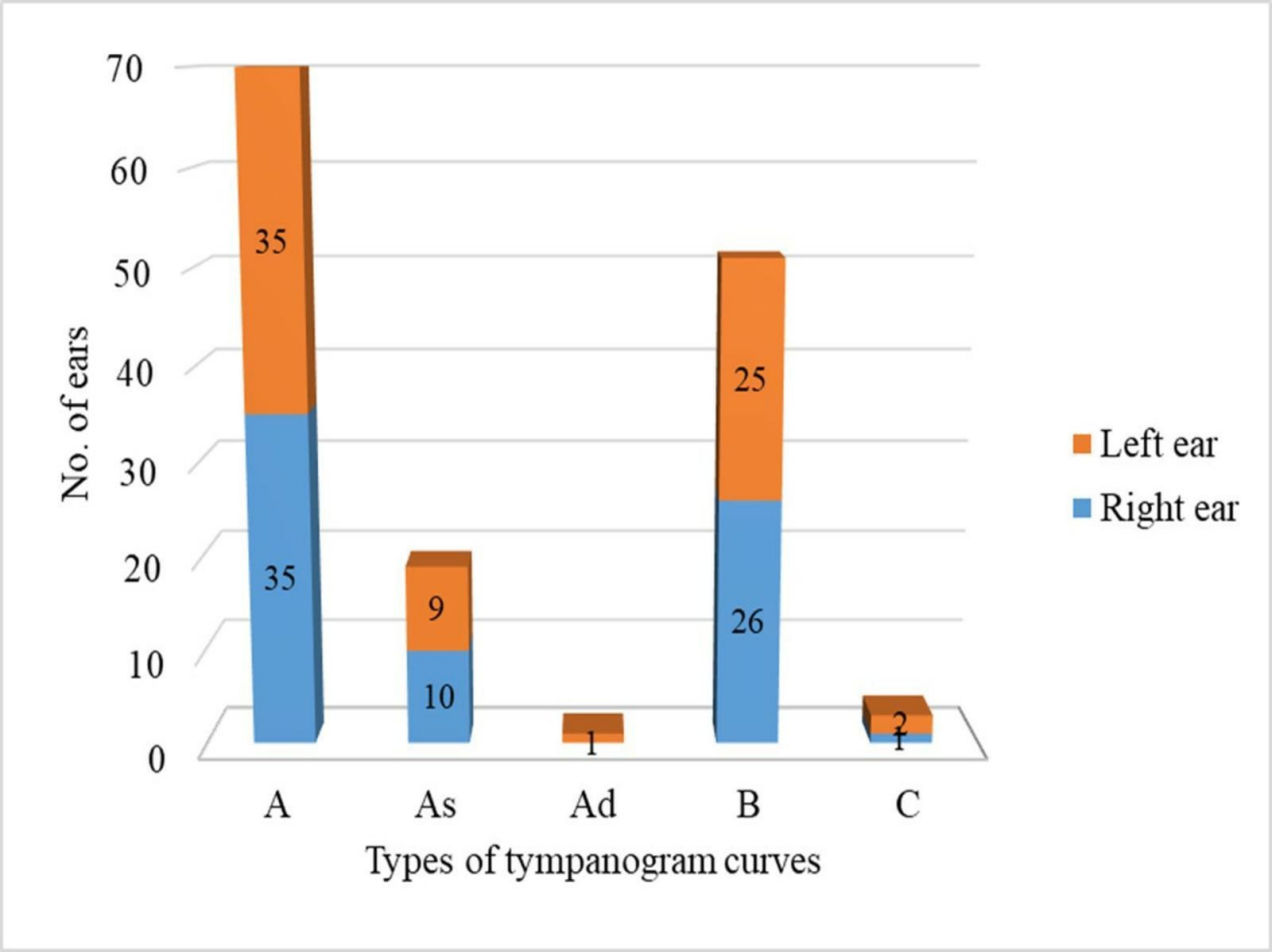
Tympanogram findings in asymptomatic children with orofacial clefts

The type A tympanogram curve was the predominant finding seen in 35 (48.6%) asymptomatic children (Figure 3).

## Discussion

Children with orofacial clefts often report to the plastic surgery OPD for intervention due to the visible deformity. However, the ear symptoms, including deafness, are often subtle and tend to be overlooked in comparison with the noticeable orofacial cleft. Surgical repair of orofacial clefts can produce satisfactory cosmetic results but the problem of otitis media with effusion (OME) persists, causing long-standing hearing loss and can affect linguistic, academic, and personal development [6-7]. Abrams et al. found a high prevalence rate of middle ear pathologies (65%) even in adults with orofacial clefts [6]. A poor Eustachian tube (ET) function, leading to reduced middle ear pressure and TM retraction, can also contribute to the increased incidence of acquired cholesteatoma in these children. Beyond the accepted explanation of ET dysfunction, potential genetic contribution to the pathogenesis of cholesteatoma has also been suggested.

Various studies have attempted to establish an association between the orofacial cleft and middle ear diseases in children [3,6]. However, the majority of these studies are retrospective studies, mostly from developed countries, and are limited by a lack of detailed information regarding the otological and audiological status of the participants. A prospective study with comprehensive data would be more accurate and reliable for this purpose. This study was from India, a developing country, and described the various ear-related problems in children with orofacial clefts. All orofacial cleft patients attending the plastic surgery clinic with or without ear complaints were also included in the study. We have, therefore, avoided selecting only those patients with ear complaints, thus providing a good estimate of the true prevalence of ear diseases in the orofacial cleft population.

According to age, the majority of the children in this study were found in the age group zero to five years. This could be attributed to the obvious deformity of the child that compelled the parents or caregivers to seek medical help early. Among 96 children with an orofacial cleft, only 24 children (25%) had a history of ear problems, whereas on ear examination, 58.3% had abnormal ear findings. This highlighted that the ear manifestations were silent and could be easily missed out unless actively looked for it.

The majority of the study population were in the age group zero to five years. The earlier age group children showed a trend of ear disease being asymptomatic compared to older children. The invisible nature of the hearing loss, with minimal discomfort to the child, could be the cause for the younger children to remain asymptomatic. It was found that older children present with more severe and symptomatic ear diseases than the younger age group. This is contrary to Gopalakrishna et al. and Sheahan et al.'s findings of improvement in middle ear diseases with increasing age due to the improvement in the ET function [8-9]. 

Significant middle ear problems were present in children in the age group zero to five years with 60% of them having some degree of hearing loss. It could be due to the fact that the majority of parents of children less than two years of age might fail to notice subtle ear problems. The predominant ear finding noted in this study was the features of MEE. This was seen in 94 ears (49%). This agrees well with the high figure of 74.7% prevalence of MEE in children with clefts in the age group from one to five years of age in a prospective study by Flynn et al. in Sweden. They reported OME as the leading cause of hearing loss in 83.1% of ears [10]. Apart from mild conductive hearing loss, MEE does not cause any other symptoms of discomfort and is therefore easily overlooked. In infants, OME combined with hearing loss may continue for weeks or even months without being detected. Early diagnosis and treatment are invaluable in preventing future hearing loss. If left untreated, this may progress to middle ear retraction and chronic otitis media.

We found that children with an isolated cleft palate and a cleft lip with palate had a higher incidence of ear problems in comparison to children with an isolated cleft lip. A higher incidence and an increase in the severity of middle ear disease may be anticipated in children with more overt palatal malformations. In our study, symptomatic ear problems were seen in only the cleft of the palate alone (group II orofacial cleft) and the cleft lip and palate (group III cleft). The more severe the orofacial cleft, the more the children have symptomatic ear problems and abnormal ear findings on examination. Similarly, Sheahan et al. reported the high prevalence (60%) of middle ear problems in subjects with different types of cleft palates with or without cleft lips compared with just 8% in patients with isolated cleft lips/alveoli [9]. This assumption is supported by the study of Schwartz et al. who reported that children with only minimal notching or a terminal slit were at a lower risk of developing otitis media than those with a severe cleft during the first year of life, emphasizing the importance of cleft severity [11]. However, some studies show no obvious relationship between the degree of clefting and otologic outcomes, including the incidence of hearing loss, the prevalence of otitis media, and the incidence of TM retraction and perforation [12-13].

In our study, three children with a submucosal cleft (37.5%) had features of MEE. Patients with submucous clefts are also at high risk of middle ear diseases like other types of overt orofacial clefts, with MEE being the most common middle ear pathology seen in different types of clefts.

In our study, normal hearing was seen in only 41.6% (80 ears) and a normal tympanogram was seen in only 45% (81 ears); the results were comparable to other studies [13-14]. Zanzi M et al. conducted a study on 18 children with cleft palates; it also showed that the impedance tympanogram and PTA results were normal in only in 22% and 27.8% of patients, respectively [15]. This emphasizes the need for a hearing evaluation in orofacial cleft patients. The prevalence of cholesteatoma in our study was 3.1% (6 children). Despite the surgical correction of clefts and the early treatment of OME, structural changes of the TM progress until the development of retraction pockets and cholesteatomas in up to 9.2% of the patients. Children with cleft palates have a 100- to 200-fold higher probability of developing cholesteatoma than children without a cleft palate [16].

Imbery et al. revealed the importance of routine follow-up care by an otolaryngologist to minimize the complications of ear disease. The rate of cholesteatoma formation was reduced from 9% to 3% when a coordinated team approach was instituted to care for the orofacial cleft patients [17]. Once again stressing on the significance of routine ear check-ups, Goudy S et al. claimed that 50% of orofacial cleft children continue to suffer from ear diseases as adults, making it a difficult situation to manage [18]. In our study, there is a trend of MEE being a predominant finding in the zero to five years age group and chronic otitis media squamosal disease being a predominant finding in the more than 10 years age group.

In the present study, various degrees of hearing loss were seen in 112 ears (58.3%) of subjects. Conductive hearing loss of up to 28 ±13.3 dB in the pure tone audiogram and 34±19 dB in BERA were noticed in the study population. Children with cleft palates may suffer recurrent or continuous OME, causing atelectasis, ossicular fixation, and tympanosclerosis, which can result in conductive hearing loss of up to 30 dB [19-20]. Luthra et al. found that the air conduction thresholds ranged from 20.37 dB to 28.89 dB in orofacial cleft children [21]. The associated hearing loss was mild and did not show much variation between the different age groups.

The predominant abnormal finding in tympanogram was the B type curve seen in 65 ears (36.1%) in our study. A study by Handzik et al. showed similar results with tympanogram curves in cleft children [22]. This high rate of the B type curve may be explained due to the presence of MEE. A thorough evaluation by an otolaryngologist should be the standard of care in the management of all children with orofacial clefts. A hearing problem in children with orofacial clefts can subsequently cause speech disorders and further disrupt the learning and development of the child.

The major limitation of this study was that it looked at the point prevalence of middle ear diseases in children with orofacial clefts. A cohort study with a larger sample size over a long duration, to study the course of the middle ear dysfunction secondary to orofacial clefts would be needed. The second limitation was that a relatively smaller number of subjects with group I, I (A), and II (S) clefts were studied. Hence, their ear findings and audiological profile could not be studied well.

## Conclusions

This descriptive study assessed one of the common, but ignored, problems encountered by otolaryngology practitioners, that is, middle ear disease in children with orofacial clefts. The results of this study once again confirmed the higher incidence of middle ear diseases, although up to half of the orofacial patients may never complain of ear problems. On the basis of this study, patients with cleft lip and palate should undergo a detailed otologic and audiologic assessment to identify any middle ear pathology and hearing loss so that timely intervention can be taken.
